# Combined Intraoperative Arthroscopic and Fluoroscopic Guided Reduction of a Lateral Tibial Plateau Fracture Using Minimally Invasive Metaphyseal and Intraarticular Fixation: Description of a Surgical Technique

**DOI:** 10.7759/cureus.15834

**Published:** 2021-06-22

**Authors:** Waleed Albishi, Abdulrahman M Alsharidah, Abdullah Alkhuraiji, Zaheer Dalati, Hisham Alsanawi

**Affiliations:** 1 Orthopedic Surgery, College of Medicine, King Saud University, Riyadh, SAU; 2 Orthopedic Surgery, King Saud University Medical City, Riyadh, SAU; 3 Orthopedic Surgery, King Saud University, Riyadh, SAU

**Keywords:** lateral tibial plateau, arthroscopic reduction, schatzker type iii, trauma, arthroscopy

## Abstract

Tibial plateau fractures are quite common among lower limb fractures*. *Several fracture classifications exist including Schatzker classification, in which tibial plateau fractures are divided into six types where each increasing numerical category indicates increasing severity of the injury and worsening prognosis. Arthroscopic-assisted techniques using a lateral or medial metaphyseal window have shown results comparable to open internal fixation methods with multiple advantages. We present a case of a medically and surgically free 40-year-old lady who presented to our emergency department complaining of left knee pain following a fall from the stairs. Clinically there was significant swelling and tenderness over the lateral aspect of the left proximal tibia, radiographs showed a Schatzker type III tibial plateau fracture, confirmed by computed tomography (CT). A combined intraoperative arthroscopic- and fluoroscopic-guided reduction of the articular depression through a lateral cortical window was achieved and the fracture was fixed using a minimally invasive fixation technique. The postoperative course was uneventful. The patient had recovered full range of motion and the wounds were barely visible. One-year X-ray showed healed fracture without any evidence of displacement or subsidence.

## Introduction

Tibial plateau fractures represent 5-8% of lower limb fractures [1]. Several fracture classifications exist including Schatzker classification, which is the most widely used classification system. He divides the tibia plateau fracture into six types: Lateral plateau split fracture (Type I), Lateral plateau split fracture with depression (Type II), Lateral plateau pure depression fracture (Type III), Medial plateau fracture (Type IV), Bicondylar fracture (Type V), and Tibial plateau fracture with diaphyseal discontinuity (Type VI). In this classification, each increasing numerical category indicates the increasing severity of the injury and worsening prognosis [2, 3]. Arthroscopic assisted techniques using a lateral or medial metaphyseal window have shown results comparable to open internal fixation methods with multiple advantages including limited dissection of the soft tissue around the fracture, direct visualizing of fracture fragments which helps ensure anatomical reduction of the articular surface, and the possibility of evaluating and treating other concomitant intraarticular pathologies [4-7]. In this paper, we describe an arthroscopic- and fluoroscopic-assisted reduction technique combined with minimally invasive metaphyseal fixation using a bioabsorbable interference screw and intraarticular fixation using percutaneous 3.5 cannulated screws in a healthy 40-year-old patient who presented with a Schatzker Type III tibial plateau fracture.

## Case presentation

**Figure 1:**
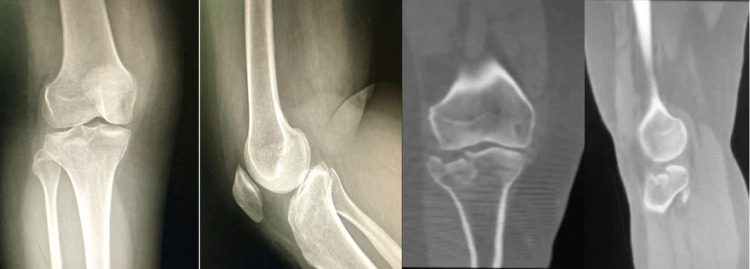
Anteroposterior and lateral X-ray along with coronal and sagittal CT scan of the knee showing a depressed lateral plateau fracture with significant displacement (Schatzker Type III tibial plateau fracture).

The patient is a 40-year-old female who presented to the emergency department with a complaint of left knee pain and swelling from falling down the stairs. She had no significant past medical or surgical history, nor a history of similar trauma. Clinical examination showed swelling of the knee and tenderness over the lateral aspect of the proximal tibia. Knee ligaments were stable and neurovascular status was intact. X-ray images showed a Schatzker Type III tibial plateau fracture. Computed tomography and magnetic resonance imaging better defined the fracture pattern and were used to rule out any concomitant knee pathologies (Figure 1). Surgery was performed after signed consent. The patient was informed that data concerning her case would be submitted for publication. Her consent was obtained.

Surgical technique

**Figure 2:**
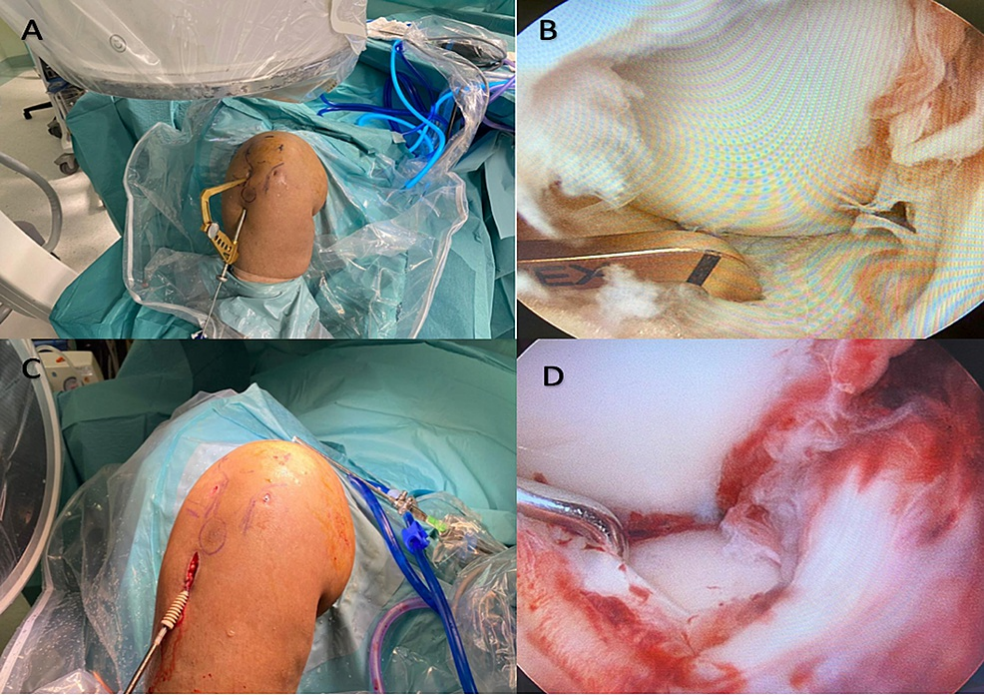
Clinical images of the right knee: A, The ACL tibia guide is used to direct the guide wire into the fractured lateral tibia plateau. B, Arthroscopic image confirming the position of the ACL tibia guide at the fractured fragment. C, Bioabsorbable interference screw is used after fracture reduction and bone grafting were done (screw size 10). D, Final arthroscopic image confirming the reduction of the fracture. ACL; anterior curciate ligament

Following a careful evaluation of the pre-operative CT scan, we planned to achieve a combined intraoperative arthroscopic- and fluoroscopic-guided reduction of the articular depression through a lateral cortical window. The fracture was fixed using a minimally invasive fixation technique. The patient was supine on a Jackson table under general anesthesia. She received 2 g of intravenous cefazolin for infection prophylaxis. Non-sterile tourniquet was applied. The right knee was prepared, draped, and flexed to 90 degrees using foot support and a lateral thigh kidney shape support. A standard lateral and medial anterior arthroscopic portals were made. Diagnostic knee arthroscopy was conducted and the fracture was assessed. No other knee pathologies were identified. The inflow pressure was kept to a minimum throughout the case to decrease the risk of fluid extravasation and reduce the subsequent potential for increased compartment pressure. An anterior cruciate ligament (ACL) guide was used to place a drill-tipped guide pin in the center of the depressed fragment through a small incision in the proximal anterolateral aspect of the tibia (Figure 2).

**Figure 3:**
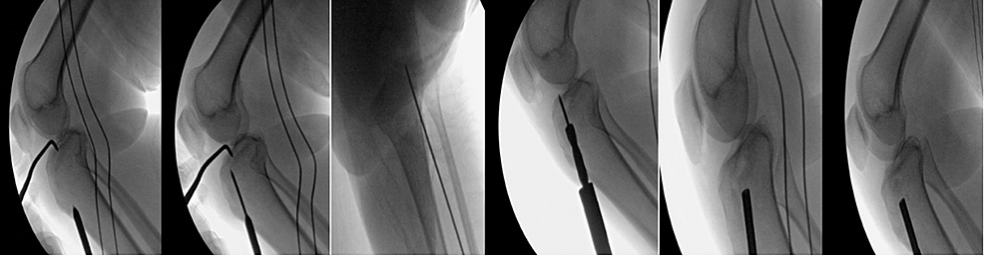
Serial of fluoroscopic images showing the surgical technique using the ACL tibia guide and reamer (size 8). A bone graft and a bone impactor were also used to achieve joint reduction. ACL; anterior curciate ligament

Intraoperative anterior-posterior (AP) and lateral fluoroscopy were used to confirm that the guidewire was drilled into the desired fragment with the appropriate trajectory depending on the direction chosen on pre-operative CT. A size 8 coring reamer was used to circumferentially open the tibial cortex while as little bone as possible was removed. A bone impactor was used to elevate the depressed fragment. The anatomical reduction was obtained and confirmed by arthroscopy and fluoroscopy (Figure 3).

**Figure 4:**
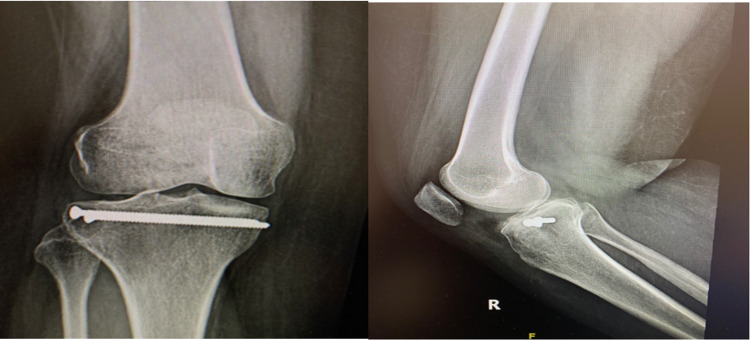
Anteroposterior and lateral X-ray showing healed fracture without any evidence of displacement or subsidence.

The resulting metaphyseal defect was grafted using bone allograft and a size 10 bioabsorbable screw was then introduced through the reamed bone tunnel to support the impacted bone below the reduced articular surface. In addition, two percutaneous 3.5 mm cortical screws were introduced subchondral from lateral to medial. Final fluoroscopic images confirmed the anatomic reduction of the fracture. The skin was closed and a dry dressing applied. The patient was allowed to have an immediate range of motion as tolerated. Partial weight-bearing was recommended initially and full weight-bearing was allowed six weeks after surgery. The postoperative course was uneventful. The patient had recovered full range of motion and the wounds were barely visible. One-year X-ray showed healed fracture without any evidence of displacement or subsidence (Figure 4).

## Discussion

The efficacy of treatment of tibial plateau fractures via arthroscopic-assisted technique is based on radiological and clinical scores, including the hospital for special surgery and Rasmussen clinical score. The established literature shows, regardless of Schatzker classification, that this technique yields excellent clinical scores in the majority of cases [8-10]. Rossi et al. found that 89% of their patient population had an excellent HSS (Hospital for Special Surgery) score for Schatzker Type II and III (11). Chan et al. had an excellent clinical and radiological score in Schatzker Type I to VI in two- and 10-year follow-up periods [8]. Van Glabbeck et al. looked at patients with Schatzker Type I to V tibial plateau fractures and found that patients with Type I and III had excellent Rasmussen scores post the arthroscopic-assisted technique [9].

When compared to other techniques, such as open-reduction, the literature demonstrates that the arthroscopic technique is superior with fewer complications. Compared to an open approach, it was found to have less soft tissue damage, better visualization of the articular surface, earlier return to physical activities, and decreases need for meniscal detachment repair [4, 11-13]. Cassard et al. found that an arthroscopic approach allowed for more rapid rehabilitation, a decrease in overall hospital length of stay, and a decrease in the rate of complications [14]. Overall, the literature establishes that the arthroscopic-assisted technique has similar and at times better clinical scores compared to the open technique [4, 11-19]. When compared to the use of fluoroscopy, in the setting of closed reduction technique, Lobenhoffer et al. demonstrated that there was no discernible difference between the two techniques [15]. However, in another study, Alvarez et al. showed that arthroscopy was better than fluoroscopy, providing greater visualization, which in turn allowed for more precise tamping and reduction of the tibial plateau [19]. Ziogas et al. reported in a case report that the use of arthroscopy in balloon osteoplasty showed an excellent clinical outcome at weeks 6, 12, and 24 post-op [20].

In our case, a combined arthroscopic- and fluoroscopic-guided approach aided in the direct and indirect assessment of the fracture fragment and led to optimal reduction of the articular surface with minimal soft tissue dissection. The use of intraoperative X-rays ensured that the path of our guidewire was at the angle and in the direction of the desired preoperative reduction plane. Using the coring reamer to create the lateral window helped to form a confined and well-supported tunnel while removing as little bone as possible. Following fracture reduction and bone grafting of the metaphyseal defect, a bioabsorbable screw 2 mm wider than the reamed tunnel helped achieve a press-fit fixation and the implant served as support under the reduced fragment to decrease the risk of subsidence. The addition of two 3.5 subchondral percutaneous screws helped add more stability to the construct and gave us the confidence to progress patient rehabilitation. Range of motion exercises was allowed from the start and the patient recovered her full knee range of motion at six weeks follow-up appointment, during which we advanced her weight-bearing status to full weight-bearing as tolerated. The patient made a full recovery without any complications. In our technique, combining intraoperative arthroscopic and fluoroscopic guided reduction and using minimally invasive metaphyseal and intraarticular fixation, has not been previously reported. We believe that the combination of all this reduction and fixation technique was needed to help accelerate the patient’s recovery so that she could return to her normal daily activity faster without the concern of reduction or fixation failure.

## Conclusions

We believe that the combined technique used helped achieve the desired surgical goals with minimal soft tissue dissection and smaller surgical incisions compared to other surgical techniques.

## References

[1] Weimann A, Heinkele T, Herbort M, Schliemann B, Petersen W, Raschke MJ (2013). Minimally invasive reconstruction of lateral tibial plateau fractures using the jail technique: a biomechanical study. BMC Musculoskelet Disord.

[2] Schatzker J, McBroom R, Bruce D (1979). The tibial plateau fracture: the Toronto experience 1968-1975. Clin Orthop Relat Res.

[3] Schatzker J (1974). Compression in the surgical treatment of fractures of the tibia. Clin Orthop Relat Res.

[4] Gill TJ, Moezzi DM, Oates KM, Sterett WI (2001). Arthroscopic reduction and internal fixation of tibial plateau fractures in skiing. Clin Orthop Relat Res.

[5] Lemon RA, Bartlett DH (1985). Arthroscopic assisted internal fixation of certain fractures about the knee. J Trauma.

[6] Burdin G (2013). Arthroscopic management of tibial plateau fractures: surgical technique. Orthop Traumatol Surg Res.

[7] Hartigan DE, McCarthy MA, Krych AJ, Levy BA (2015). Arthroscopic-assisted reduction and percutaneous fixation of tibial plateau fractures. Arthrosc Tech.

[8] Chan YS, Chiu CH, Lo YP, Chen AC, Hsu KY, Wang CJ, Chen WJ (2008). Arthroscopy-assisted surgery for tibial plateau fractures: 2- to 10-year follow-up results. Arthroscopy.

[9] van Glabbeek F, van Riet R, Jansen N, D'Anvers J, Nuyts R (2002). Arthroscopically assisted reduction and internal fixation of tibial plateau fractures: report of twenty cases. Acta Orthop Belg.

[10] Rivera F, Bianciotto A (2020). Contraceptive subcutaneous device migration: what does an orthopaedic surgeon need to know? A case report and literature review. Acta Biomed.

[11] Rossi R, Bonasia DE, Blonna D, Assom M, Castoldi F (2008). Prospective follow-up of a simple arthroscopic-assisted technique for lateral tibial plateau fractures: results at 5 years. Knee.

[12] Duan XJ, Yang L, Guo L, Chen GX, Dai G (2008). Arthroscopically assisted treatment for Schatzker type I-V tibial plateau fractures. Chin J Traumatol.

[13] Egol KA, Cantlon M, Fisher N, Broder K, Reisgo A (2017). Percutaneous repair of a schatzker iii tibial plateau fracture assisted by arthroscopy. J Orthop Trauma.

[14] Cassard X, Beaufils P, Blin JL, Hardy P (1999). [Osteosynthesis under arthroscopic control of separated tibial plateau fractures. 26 case reports]. Rev Chir Orthop Reparatrice Appar Mot.

[15] Lobenhoffer P, Schulze M, Gerich T, Lattermann C, Tscherne H (1999). Closed reduction/percutaneous fixation of tibial plateau fractures: arthroscopic versus fluoroscopic control of reduction. J Orthop Trauma.

[16] Kayali C, Oztürk H, Altay T, Reisoglu A, Agus H (2008). Arthroscopically assisted percutaneous osteosynthesis of lateral tibial plateau fractures. Can J Surg.

[17] Shen G, Zhou J (2011). [Comparison study on effectiveness between arthroscopy assisted percutaneous internal fixation and open reduction and internal fixation for Schatzker types II and III tibial plateau fractures]. Zhongguo Xiu Fu Chong Jian Wai Ke Za Zhi.

[18] Zhou Z (2009). [Arthroscopic percutaneous osteosynthesis of low-energy tibial plateau fractures]. Zhongguo Xiu Fu Chong Jian Wai Ke Za Zhi.

[19] Alvarez A, Youn GM, Remigio Van Gogh AM, Shin Yin SS, Chakrabarti MO, McGahan PJ, Chen JL (2020). Tibial plateau with arthroscopic reduction-internal fixation. Arthrosc Tech.

[20] Ziogas K, Tourvas E, Galanakis I, Kouvidis G (2015). Arthroscopy assisted balloon osteoplasty of a tibia plateau depression fracture: a case report. N Am J Med Sci.

